# Respective contribution of baseline clinical data, tumour metabolism and tumour blood-flow in predicting pCR after neoadjuvant chemotherapy in HER2 and Triple Negative breast cancer

**DOI:** 10.1186/s13550-024-01115-4

**Published:** 2024-07-04

**Authors:** Neree Payan, Benoit Presles, Charles Coutant, Isabelle Desmoulins, Sylvain Ladoire, Françoise Beltjens, François Brunotte, Jean-Marc Vrigneaud, Alexandre Cochet

**Affiliations:** 1 Department of Nuclear Medicine, Georges-François Leclerc Cancer Centre, Dijon, France; 2 IFTIM, ICMUB Laboratory, UMR CNRS 6302, University of Burgundy, Dijon, France; 3 Department of Medical Oncology, Georges-François Leclerc Cancer Centre, Dijon, France; 4 Department of Tumor Biology and Pathology, Georges-François Leclerc Cancer Centre, Dijon, France

**Keywords:** Breast cancer, Blood flow, Texture Features, Pathological Response, ^18^F-FDG PET/CT

## Abstract

****Background**:**

The aim of this study is to investigate the added value of combining tumour blood flow (BF) and metabolism parameters, including texture features, with clinical parameters to predict, at baseline, the pathological complete response (pCR) to neoadjuvant chemotherapy (NAC) in patients with newly diagnosed breast cancer (BC).

****Methods**:**

One hundred and twenty-eight BC patients underwent a ^18^F-FDG PET/CT before any treatment. Tumour BF and metabolism parameters were extracted from first-pass dynamic and delayed PET images, respectively. Standard and texture features were extracted from BF and metabolic images. Prediction of pCR was performed using logistic regression, random forest and support vector classification algorithms. Models were built using clinical (C), clinical and metabolic (C+M) and clinical, metabolic and tumour BF (C+M+BF) information combined. Algorithms were trained on 80% of the dataset and tested on the remaining 20%. Univariate and multivariate features selections were carried out on the training dataset. A total of 50 shuffle splits were performed. The analysis was carried out on the whole dataset (HER2 and Triple Negative (TN)), and separately in HER2 (N=76) and TN (N=52) tumours.

****Results**:**

In the whole dataset, the highest classification performances were observed for C+M models, significantly (*p*-value<0.01) higher than C models and better than C+M+BF models (mean balanced accuracy of 0.66, 0.61, and 0.64 respectively). For HER2 tumours, equal performances were noted for C and C+M models, with performances higher than C+M+BF models (mean balanced accuracy of 0.64, and 0.61 respectively). Regarding TN tumours, the best classification results were reported for C+M models, with better performances than C and C+M+BF models but not significantly (mean balanced accuracy of 0.65, 0.63, and 0.62 respectively).

****Conclusion**:**

Baseline clinical data combined with global and texture tumour metabolism parameters assessed by ^18^F-FDG PET/CT provide a better prediction of pCR after NAC in patients with BC compared to clinical parameters alone for TN, and HER2 and TN tumours together. In contrast, adding BF parameters to the models did not improve prediction, regardless of the tumour subgroup analysed.

**Supplementary Information:**

The online version contains supplementary material available at 10.1186/s13550-024-01115-4.

## Introduction

Breast cancer (BC) is the most commonly diagnosed cancer in women worldwide [1], and its global burden continues to increase [2, 3]. This heterogeneous disease is composed of various molecular subtypes with different histological characteristics, treatment strategies, aggressiveness, and outcomes [4, 5]. Neoadjuvant chemotherapy (NAC) is a standard treatment approach for locally advanced BC for which a direct radical resection is not preferred. The main advantage of NAC lies in its ability to reduce tumour size and downgrade its burden to facilitate breast-conserving surgery. Pathological complete response (pCR) to NAC has been identified as a surrogate of potential interest for treatment outcome, especially in aggressive BC subtypes, for which strong associations with long-term event-free and overall survival have been reported [6, 7]. Conversely, in aggressive BC subtypes, a non-response to NAC (non-pCR) is associated with higher risks of relapse and lower survival. Depending on the BC subtype, only 5 to 50% of patients achieve a pCR. NAC may also be limited by toxic side-effects that can persist over time affecting patients’ daily life [8]. Therefore, improvements in early prediction of NAC response remain of paramount importance, to prevent/limit toxicities caused by an ineffective treatment and to facilitate personalised medicine.

Many studies have been carried out to predict the histopathological response of BC to NAC. Approaches based on Positron Emission Tomography/Computed Tomography (PET/CT) have shown high predictive value when studying metabolic response during treatment [9–11]. Few studies have also considered tumour perfusion evaluation for monitoring BC therapies and evaluated its ability to provide predictive and prognostic information [12–14]. As discrepancies between tumour metabolism and blood flow (BF) have been reported in the literature [15], the rationale behind evaluating these two biomarkers combined lies in the idea of enhancing their individual performances in BC characterisation and prognostic stratification. In addition, the interest in advanced quantitative imaging data, such as texture analysis (TA), has also increased significantly in BC research [16, 17], providing new insights into the global heterogeneity of tumours in a non-invasive manner [18]. Improved NAC response prediction has been reported when clinical, morphological and metabolic data have been combined with metabolic-based textural information [19–21].

As baseline studies may be preferable with the idea of selecting patients with the highest chance of a good response before starting chemotherapy, several studies have focused on baseline parameters [19–22]. These studies often suffer from the drawback of mixing the three main histological subtypes of breast cancer: HER2, Triple Negative (TN) and luminal. Indeed, the pCR has a very low rate in the luminal subtype, making other criteria less predictive in this subtype. Parameters used for prediction of the response to NAC also differ from one study to another, and the number of patients included is sometimes very limited. To address some of these limitations, the present study is based on a significant number of patients, which is allowed by the size of our cohort, to exclude luminal tumours and to clearly separate HER2 and TN breast cancers. Different models will be built and compared considering various sets of baseline clinical and histopathological parameters, global and textural metabolic and BF PET information, for pCR prediction in newly diagnosed localised BC.

## Material and methods

### Patient characteristics and pathological analysis

The present cohort of patients has already been described in detail in previous publications from our institution [23]. To summarise, a total of 217 patients with newly diagnosed stage II or III breast cancer and with an indication for NAC were prospectively considered for participation to this study. Based on oncologists’ recommendations, these patients were recruited from July 2011 to May 2017 for a ^18^F-Fluorodeoxyglucose (FDG) PET/CT scan before treatment. Patients with high glycaemia (> 9 mmol/L) and unable or unwilling to undergo PET scans were excluded. The classification of malignant tumours (TNM) was established in accordance with the 8^th^ edition of the American Joint Committee on Cancer (AJCC) staging system [24]. Histological characteristics, including estrogen receptor (ER), progesterone receptor (PR) and HER2 expression, were assessed from core biopsies of primary tumours. ER and PR status were considered positive if tumours showed at least 10% of positive cells. HER2 status was assessed by immunohistochemistry or fluorescent in situ hybridisation (FISH) following the ASCO/CAP recommendations, and graded from 0 to 3+, with scores 3+ considered as positive. In ambiguous cases (2+), HER2 amplification confirmed by FISH was used to assess positivity. Patients were classified into three groups according to their molecular subtypes: HER2 (HER2-amplified), Luminal (hormone receptors (HR)-positive, HER2 non-amplified) and TN (HR-negative, HER2 non-amplified). Seventy-nine patients were HER2 amplified (36.4%), 81 were HR+/HER2- (37.3%) and 57 (26.3%) had TN tumours. The institutional review board approved this prospective study as a current care study (NCT02386709). The patient’s non-opposition was documented in source documents by the medical team and used as the patient’s informed consent.

### Treatment and clinical endpoint

Women with human epidermal growth factor receptor 2 (EGFR2) HER2 amplified tumours were treated with trastuzumab and docetaxel-based regimens. Women with TN or luminal/HER2 non-amplified tumours received six sequential chemotherpay with anthracyclines and taxanes, or six cycles of FEC100 (epirubicin 100 mg/m^2^, 5-fluorouracil 500 mg/m^2^, and cyclophosphamide 500 mg/m^2^). Tumours were surgically removed within one month after the last course of chemotherapy and pathological responses were assessed on the resected tumoral tissue. The Chevallier classification [25] was used to assess pathological response, with a complete response defined as the absence of residual invasive cancer after NAC at both, the breast and lymph node levels (Chevallier classification grade 1 and 2). Four patients received a non-conventional treatment (bevacizumab, paclitaxel carboplatin) and were excluded from the current analysis, leading to 213 patients.

**Fig. 1:**
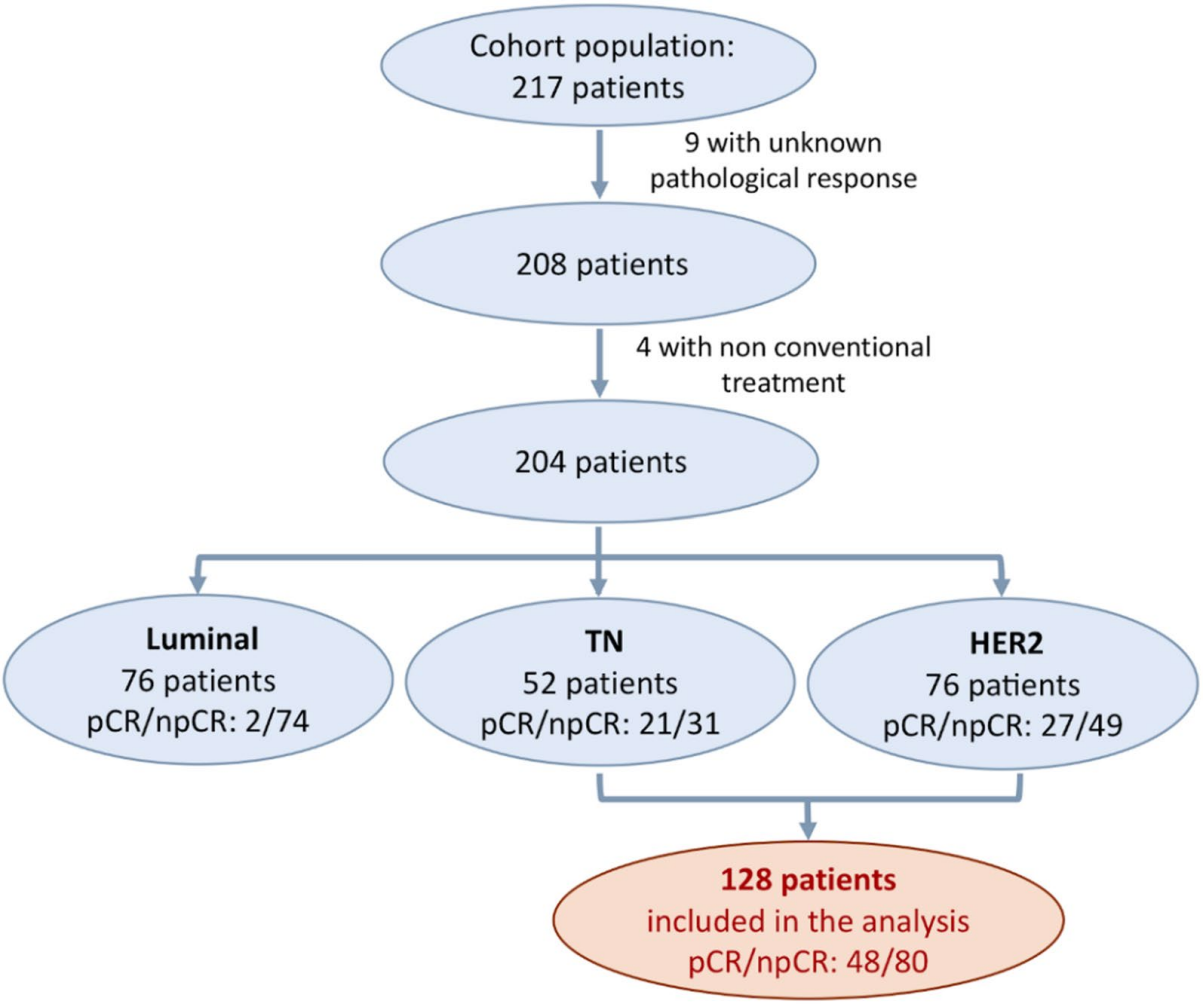
Flow chart of the final cohort. pCR: pathological complete response, npCR: non-pCR

Fifty of these patients reached a pCR, 154 were non-responders and the pathological response was unknown for nine patients. Considering that pCR is a less relevant surrogate marker of survival for the Luminal tumours, only the HER2 and TN were included in this study. A total of 128 patients with a known pathological response to NAC were included in the analysis (Fig. 1), with a median age of 48 years. All patient and tumour characteristics are summarised in Table 1.

**Table 1:** Patients and tumours characteristics

	Number of patients (%)		Median
	n=128		(± interquartile range)
Menopause		Perfused volume (mL)	
Yes	76 (59.4)	Entire population	4.5 (±9.8)
No	52 (40.6)	HER2	3.9 (±6.6)
T Stage		TN	6.6 (±20.0)
T1-2	106 (82.8)	BF_max_ (mL/min/g)	
T3-4	22 (17.2)	Entire population	0.34 (±0.28)
N Stage		HER2	0.29 (±0.28)
N0	40 (31.2)	TN	0.35 (±0.24)
N1-3	88 (68.8)	BF_mean_ (mL/min/g)	
SBR		Entire population	0.16 (±0.12)
Grade 1-2	61 (47.7)	HER2	0.16 (±0.12)
Grade 3	67 (52.3)	TN	0.17 (±0.1)
Histological type		MATV (mL)	
Ductal	126 (98.4)	Entire population	5.3 (±7.2)
Lobular	2 (1.6)	HER2	4.5 (±6.3)
ER status		TN	6.9 (±19.4)
Positive	43 (33.6)	SUV_max_	
Negative	85 (66.4)	Entire population	10.8 (±7.8)
PR status		HER2	9.1 (±7.9)
Positive	28 (21.8)	TN	12.0 (±9.5)
Negative	100 (78.1)	SUV_mean_	
Breast cancer sub-group		Entire population	6.7 (±4.3)
HER2	76 (59.4)	HER2	5.8 (±4.6)
TN	52 (40.6)	TN	7.0 (±4.5)
Pathological complete response		TLG	
pCR	48 (37.5)	Entire population	31.8 (±63.8)
npCR	80 (62.5)	HER2	26.2 (±35.0)
		TN	53.2 (±147.5)

*BF* blood flow, *ER* estrogen receptor, *PR* progesterone receptor

### PET/CT images acquisition and reconstruction

Baseline ^18^F-FDG PET/CT scans were acquired using a Gemini TruFlight PET/CT scanner (Philips Medical Systems, Eindhoven, The Netherlands), with an axial field of view of 18 cm and a transaxial slice thickness of 4 mm. The injection of a bolus of 3 MBq/kg of ^18^F-FDG was performed with an automatic PET infusion system (Bayer Medical Care, Inc., Indianola, PA, USA). Simultaneously, a first 8-min list-mode ^18^F-FDG PET scan was acquired in a prone position, centred on the breast. A low-dose CT scan of the same region was carried out for anatomical registration and attenuation correction (120 kVp, automatic tube current modulation, 0.5-s rotation time, 16 $\times$ 1.5 mm collimation, a pitch of 0.7). A dynamic first-pass image was reconstructed based on the twelve 10-s frames extracted from the first two minutes of the early static PET acquisition. Whole-body emission and transmission scans were acquired 60 min later in a supine position, followed by a delayed two-step PET/CT scan restricted to the chest, acquired in a prone position 90 min after injection. The latter was performed with 4 min per bed position. All PET images were reconstructed using a first 3D-ordered subset iterative (OSEM) time-of-flight reconstruction technique (three iterations and 33 subsets), with image matrix sizes of 144$\times$144 and 4 mm isotropic voxels. Emission data were corrected for random coincidences, decay, dead time, scatter and attenuation.

### Clinical, blood flow and metabolic data

**Fig. 2:**
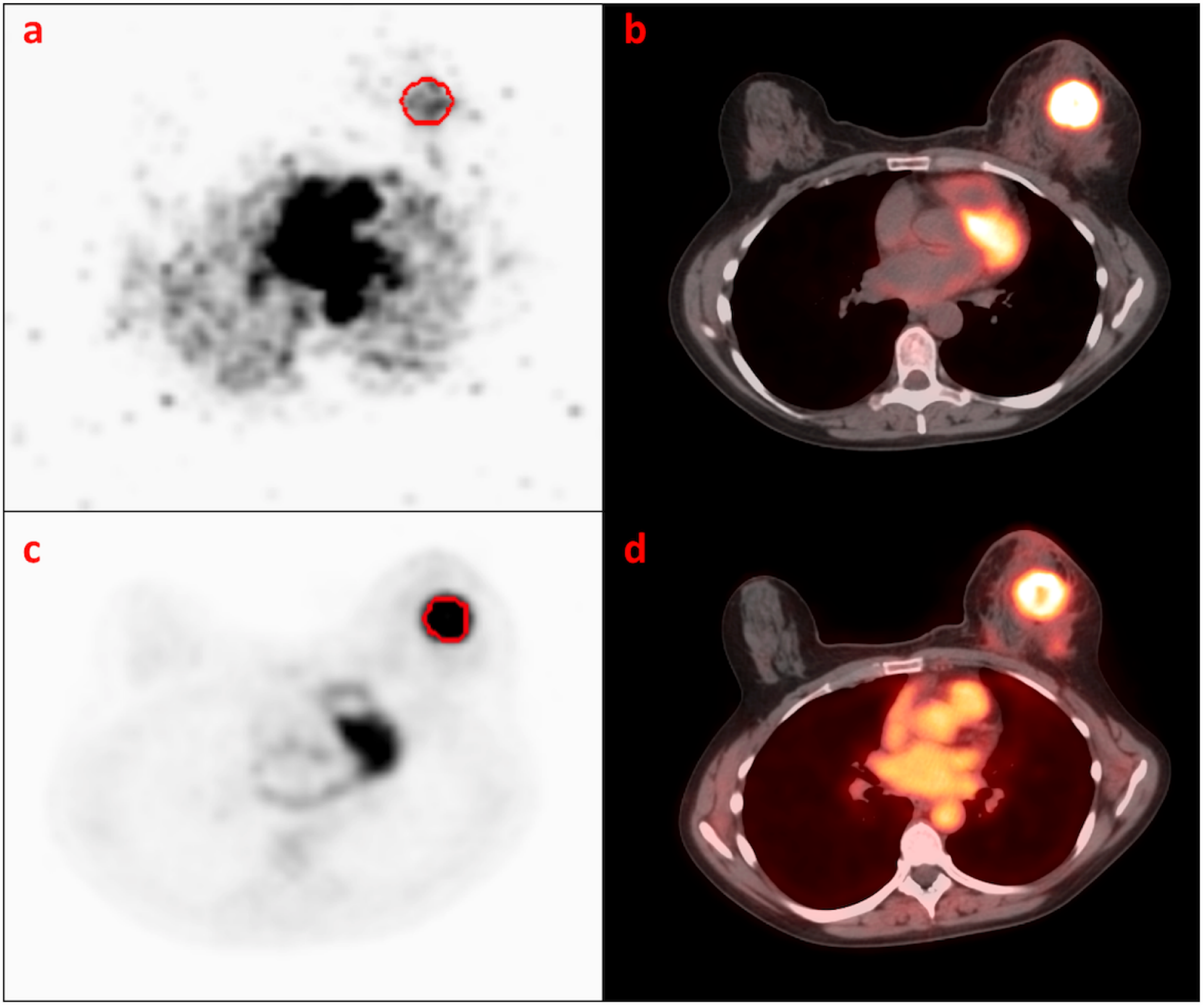
PET images of a 54-year-old woman with a T2-N1-M0 TN (HR-negative, HER2 non-amplified) ductal carcinoma. **a** parametric blood flow (mL/min/g) image from ^18^F-FDG PET first-pass dynamic (10-s frames) acquisition, **b** fusion of the first 8-min list-mode ^18^F-FDG PET and the associated CT, from which the dynamic first-pass image is reconstructed, **c** SUV parametric image from delayed (90 min post injection, 4-min per bed position) acquisition, **d** fusion of the delayed PET aquisition (Bq/mL) and the associated CT. The tumour was manually delineated on the blood flow image (**a**) and using a semi-automatic algorithm based on a contrast-dependent method [33] on the SUV parametric image (**c**)

The full methodology of the blood flow (BF) and standardised uptake value (SUV) parametric images computation has been reported in detail in a previous paper from our group [23], and is summarised in section 1 of the supplementary material. An example of parametric SUV and BF images is illustrated in Figure 2. The conventional and textural PET parameters that have been extracted from both PET parametric images are listed in section 2 of the supplementary material. All texture features (TFs) were calculated from 13 grey-level co-occurrence matrix (GLCM), one for each spatial direction, and averaged to obtain the final TFs values. Before calculating these texture parameters, it is recommended to discretise the intensities within the images [26]. For PET imaging, two main methods have been reported in the Image Biomarker Standardisation Initiative (IBSI) [27]: the absolute rescaling (AR) and the relative rescaling (RR). The rescaling method has an impact on the intensity distributions and therefore on the texture feature values, which may potentially influence the clinical interpretation [26, 28–30]. Following the same approach as in a previous work from our group [31], the two methods were applied in the present analysis using either a fixed number of 64 bins (RR) or a fixed bin size (AR) of 0.016 mL/min/g for blood flow and 0.47 SUV for metabolism [31]. All TFs were computed using in-house tools based on the Insight Segmentation and Registration Toolkit (ITK) library [32] (IBSI compliant) and are available at https://github.com/benpresles/vv. Clinical and histopathological data that were also collected and incorporated into the classification models are listed in section 3 of the supplementary material. PET data and biomarker computation characteristics are summarised in Figures S1 and S2 in supplementary material.

### Pathological Complete Response (pCR) prediction

The analysis was carried out using the entire dataset (HER2 and TN) and was also performed on the HER2 and TN subgroups independently. Patients were classified as either responders (pCR) or non-responders (non-pCR) according to the Chevallier classification. The population studied was first randomly divided into independent training (80%) and test (20%) datasets using a stratified partitioning associated with the endpoint. The training set was used for the feature selection, the optimisation and the validation of the classification models. The test set was used as an independent set to evaluate the performance of the models.

In order to evaluate the added value of blood-flow and metabolic parameters in predicting pCR after NAC, three types of models were compared:

- models using only the clinical features (C),
- models using the clinical and metabolic features combined (C+M),
- models using the clinical, metabolic and blood-flow features combined (C+M+BF).

**Fig. 3:**
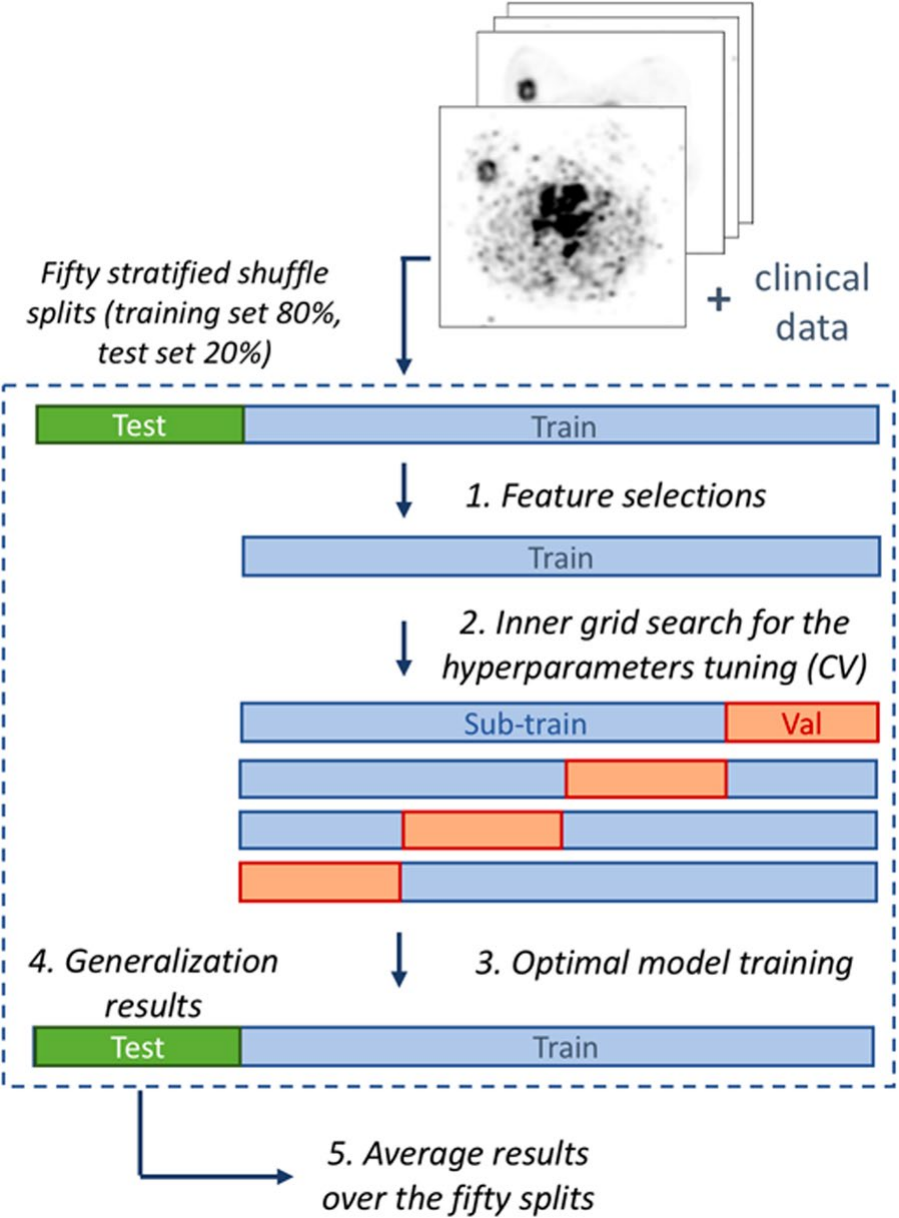
Summary of the classification machine learning pipeline. CV: cross validation, Val: validation set, Sub-train: inner training set

To assess the global additional value of these different types of data and to avoid an initial favourable/unfavourable split for one type of data, the whole process (feature selection - model optimisation - model validation) was repeated 50 times using stratified shuffle splits, as illustrated in Fig. 3.

All final model performances were assessed on the independent test dataset (Fig. 3). The performance of each model was evaluated using the balanced accuracy (BAcc). The overall performances of each model studied (C, C+M and C+M+BF) were assessed by comparing the mean values of the metrics over the 50 shuffle splits. Two-sided Mann–Whitney tests were performed between the mean balanced accuracy of each best model (C, C+M and C+M+BF), considering a *p*-value less than 0.05 as significance criteria. The features selected for every model were collected and a majority voting among the 50 splits was carried out in order to report all the features that were selected in more than 50% of the splits. These features will therefore be of interest for predicting the therapeutic response, without however providing the information on their correlations with the response status (non-response or complete response). Models using “dummies” predictions were also generated, considering “dummy” classifiers that will predict only the most frequent class label (pCR or non-pCR). The models’ performances were compared with the Dummies’ predictions. Additional classification metrics were reported: the Area Under the receiver operating characteristic Curve (AUC), the Recall, the Precision, the F1-score (which is the harmonic mean of precision and recall), and the Matthews Correlation Coefficient (MCC). The results were reported for a hard cut-off of 0.5 and assuming pCR prevalence of 37,5%, 40,4% and 35,5% (corresponding to the percentage of pCR among the known pathological responses) for the whole population (HER2 and TN), HER2 and TN subgroups, respectively.

All the machine learning pipelines characteristics, including feature selection and models optimisation, are reported in Figure S3 and section 4 of the supplementary material. Model developments and evaluations were performed using Python v3.10.

## Results

### Classification performances – the whole population (HER2 and TN)

**Fig. 4:**
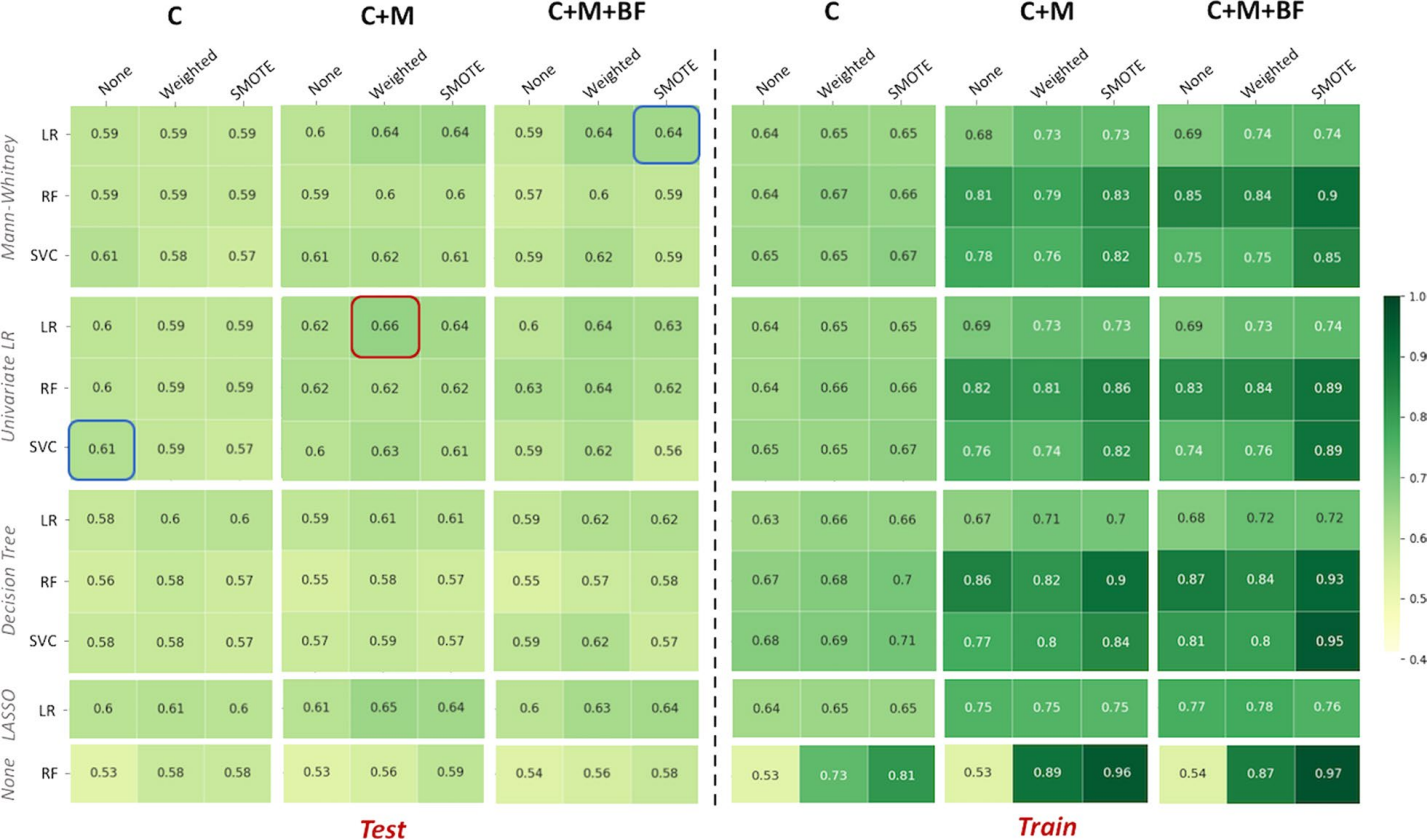
Summary of the mean balanced accuracy over the 50 shuffle splits for the test (n=26) and training (n=102) sets, for each subgroup (C, C+M and C+M+BF) among the HER2 and TN tumours combined. The best overall performance is highlighted in red; the other two highest results are highlighted in blue

When the texture information was extracted after an AR of the intensity discretisation, the best classification performance was observed for C+M models, which performed better than the best C models with significantly (*p*-value$=$0.003) higher mean balanced accuracy (mean BAcc of 0.66 vs. 0.61 respectively) and higher scores than C+M+BF models, but not significantly. These best C+M models were reported using logistic regression (LR) for feature selection and classification on weighted data. When BF information was added, the best performance was obtained using Mann Whitney (MW) as the feature selection and LR as the classifier, enhanced by data augmentation using syntactic minority oversampling technique (SMOTE) (mean BAcc = 0.64). Using clinical data only, the best results were obtained using LR as the feature selection and support vector classification (SVC) as the classifier, as shown in Figure 4.

**Table 2:** Averages over the 50 shuffle splits of the best test and training model performances for each subgroup (C, C+M and C+M+BF) for the HER2 and the TN tumours combined

	C		C+M		C+M+BF		Dummy Classifier	
	Test	Train	Test	Train	Test	Train	Test	Train
BAcc	0.61	0.65	0.66	0.73	0.64	0.74	0.50	0.50
	$(\pm 0.08)$	$(\pm 0.03)$	$(\pm 0.09)$	$(\pm 0.03)$	$(\pm 0.08)$	$(\pm 0.03)$		
F1-score	0.49	0.54	0.58	0.66	0.57	0.68	0	0
	$(\pm 0.14)$	$(\pm 0.09)$	$(\pm 0.12)$	$(\pm 0.04)$	$(\pm 0.11)$	$(\pm 0.03)$		
AUC	0.61	0.65	0.66	0.73	0.64	0.74	0.50	0.50
	$(\pm 0.08)$	$(\pm 0.03)$	$(\pm 0.09)$	$(\pm 0.03)$	$(\pm 0.08)$	$(\pm 0.03)$		
MCC	0.24	0.30	0.32	0.44	0.28	0.47	0	0
	$(\pm 0.16)$	$(\pm 0.06)$	$(\pm 0.18)$	$(\pm 0.06)$	$(\pm 0.16)$	$(\pm 0.05)$		
Recall	0.45	0.51	0.63	0.72	0.62	0.74	0	0
	$(\pm 0.15)$	$(\pm 0.10)$	$(\pm 0.16)$	$(\pm 0.04)$	$(\pm 0.16)$	$(\pm 0.04)$		
Precision	0.55	0.57	0.56	0.62	0.54	0.63	0	0
	$(\pm 0.15)$	$(\pm 0.09)$	$(\pm 0.11)$	$(\pm 0.04)$	$(\pm 0.09)$	$(\pm 0.04)$		

*BAcc* Balanced Accuracy, *MCC* Matthews Correlation Coefficient, *AUC* Area Under the ROC Curve

**Table 3:** Features selected in more than 50% of the shuffle splits of the best models for each subgroup (C, C+M and C+M+BF) when analysing HER2 and TN tumours combined

C	C+M	C+M+BF
SBR, SBR_Mitose, ER	M_entropy, M_correlation, SBR, SBR_Mitose, ER	MATV, BF_skewness, BF_correlation, BF_clustershade, N_Stage, SBR, SBR_Mitose, ER

*BF* Blood Flow, *ER* Estrogen Receptor, *M* Metabolism, *MATV* Metabolic Active Tumour Volume, *SBR* Scarff-Bloom-Richardson

All these results are summarised in Table 2. All the best models showed better classification performance than the dummies’ models. The features selected in more than half of the splits for each of the best model configurations are reported in Table 3.

When the classification was performed using TFs computed from PET images discretised with a RR, the overall performance was slightly lower, with the highest mean balanced accuracy of 0.64 for C+M+BF models, as shown in Figure S4 in supplementary material. The corresponding results for all the best models are summarised in Table S1, and the associated selected features are listed in Table S2 in the supplementary material.

### Classification performances – HER2

**Fig. 5:**
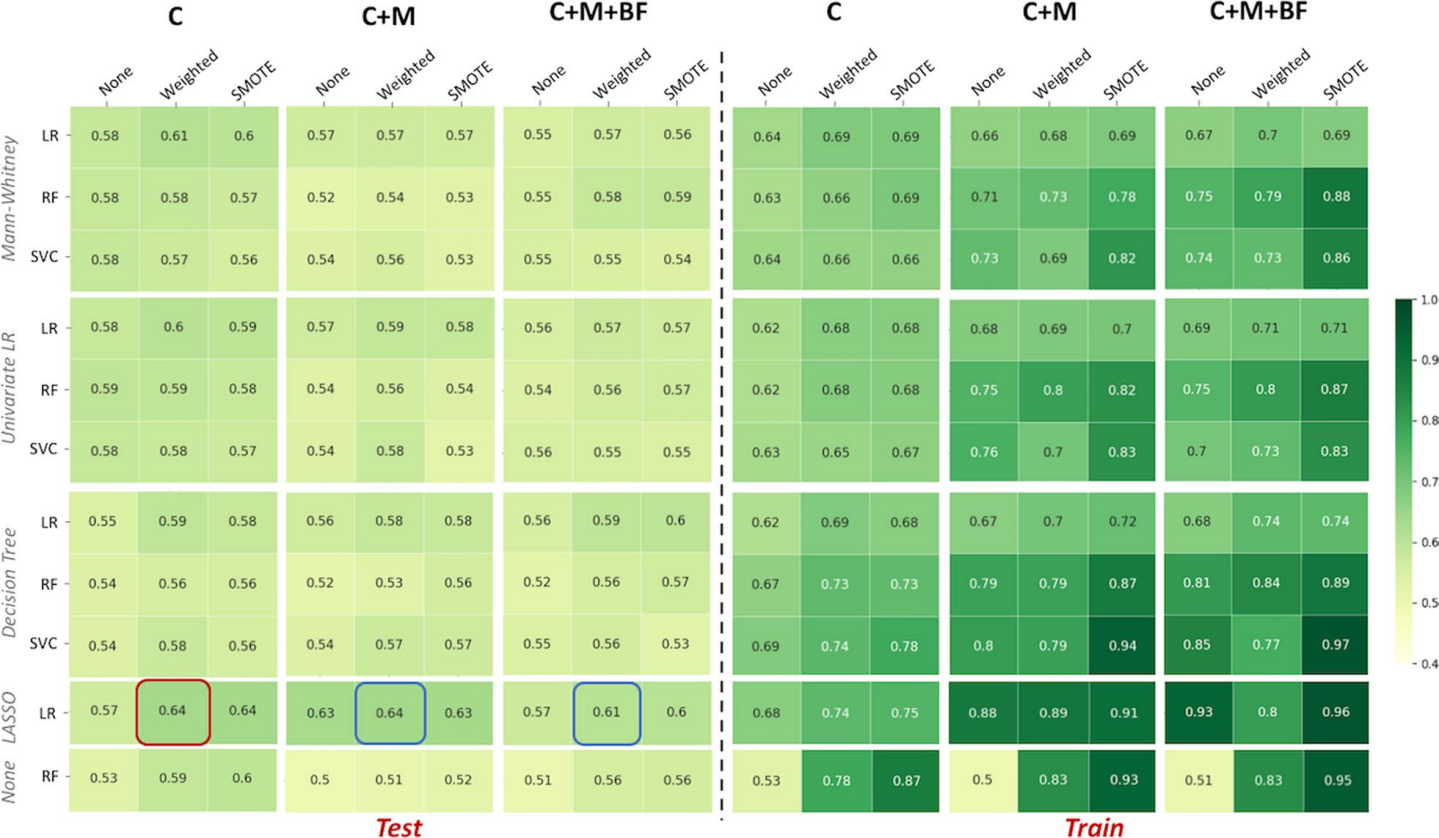
Summary of the mean balanced accuracy over the 50 shuffle splits for the test (n=15) and training (n=61) sets, for each subgroup (C, C+M and C+M+BF) among the HER2 tumours. The best overall performance is highlighted in red; the other two highest results are highlighted in blue

When only HER2 tumours were analysed, using an AR for PET image intensity discretisation, the best performance was observed equally for C and C+M models using LR classifiers with the least absolute shrinkage and selection operator (LASSO) penalisation for feature selection, on weighted data, with a mean balanced accuracy of 0.64, as shown in Figure 5.

A lower performance was recorded for the C+M+BF models, with a maximum mean balanced accuracy of 0.61, using the LR classifier with LASSO feature selection, on weighted data. No significant differences were noted between these models. Performances of these best models are reported in Table 4. All the models studied presented better classification performances than dummies’ models. The features selected in more than half of the splits for each of these models’ configurations are reported in Table 5.

When using a RR for the PET image intensity processing, the best performance was noted for C+M+BF models, with a mean BAcc$=$0.66 (Figure S5 in supplementary material). The corresponding results for all the best models, including the selected features, are presented in supplementary Table S3 and S4.

**Table 4:** Averages over the 50 shuffle splits of the best test and training model performances for each subgroup (C, C+M and C+M+BF) for the HER2 tumours

	C		C+M		C+M+BF		Dummy Classifier	
	Test	Train	Test	Train	Test	Train	Test	Train
BAcc	0.64	0.74	0.64	0.89	0.61	0.80	0.50	0.50
	$(\pm 0.12)$	$(\pm 0.04)$	$(\pm 0.12)$	$(\pm 0.08)$	$(\pm 0.14)$	$(\pm 0.04)$		
F1-score	0.55	0.66	0.55	0.84	0.50	0.74	0	0
	$(\pm 0.16)$	$(\pm 0.04)$	$(\pm 0.15)$	$(\pm 0.10)$	$(\pm 0.18)$	$(\pm 0.05)$		
AUC	0.64	0.74	0.64	0.89	0.61	0.80	0.50	0.50
	$(\pm 0.12)$	$(\pm 0.04)$	$(\pm 0.12)$	$(\pm 0.08)$	$(\pm 0.14)$	$(\pm 0.04)$		
MCC	0.29	0.46	0.29	0.75	0.22	0.58	0	0
	$(\pm 0.24)$	$(\pm 0.07)$	$(\pm 0.24)$	$(\pm 0.16)$	$(\pm 0.27$	$(\pm 0.08)$		
Recall	0.58	0.73	0.57	0.90	0.52	0.82	0	0
	$(\pm 0.20)$	$(\pm 0.07)$	$(\pm 0.19)$	$(\pm 0.10)$	$(\pm 0.22)$	$(\pm 0.06)$		
Precision	0.56	0.61	0.56	0.79	0.51	0.67	0	0
	$(\pm 0.16)$	$(\pm 0.04)$	$(\pm 0.18)$	$(\pm 0.11)$	$(\pm 0.17)$	$(\pm 0.06)$		

*BAcc* Balanced Accuracy, *MCC* Matthews Correlation Coefficient, *AUC* Area Under the ROC Curve

**Table 5:** Features selected in more than 50% of the shuffle splits of the best models for each subgroup (C, C+M and C+M+BF) when analysing HER2 tumours

C	C+M	C+M+BF
T_stage, N_stage, SBR, SBR_Mitose, Menopause, Pluri-focal, ER, Treatment	M_sd, TLG, M_skewness, M_kurtosis, TLG, M_energy, M_entropy, M_IDM, M_clustershade, Age, T_stage, N_stage, SBR, SBR_Mitose, Menopause, Pluri-focal, ER, PR, Treatment	M_skewness, M_sd, M_energy, BF_energy, BF_IDM, T_stage, N_stage, SBR, SBR_Mitose, Pluri-focal, ER, Treatment

*BF* Blood Flow, *IDM* Inverse Different Moment, *M* Metabolism, *ER* Estrogen Receptor, *PR* Progesterone Receptor, *TLG* Tumour Lesion Glycolysis, *SBR* Scarff-Bloom-Richardson

### Classification performances – TN

**Fig. 6:**
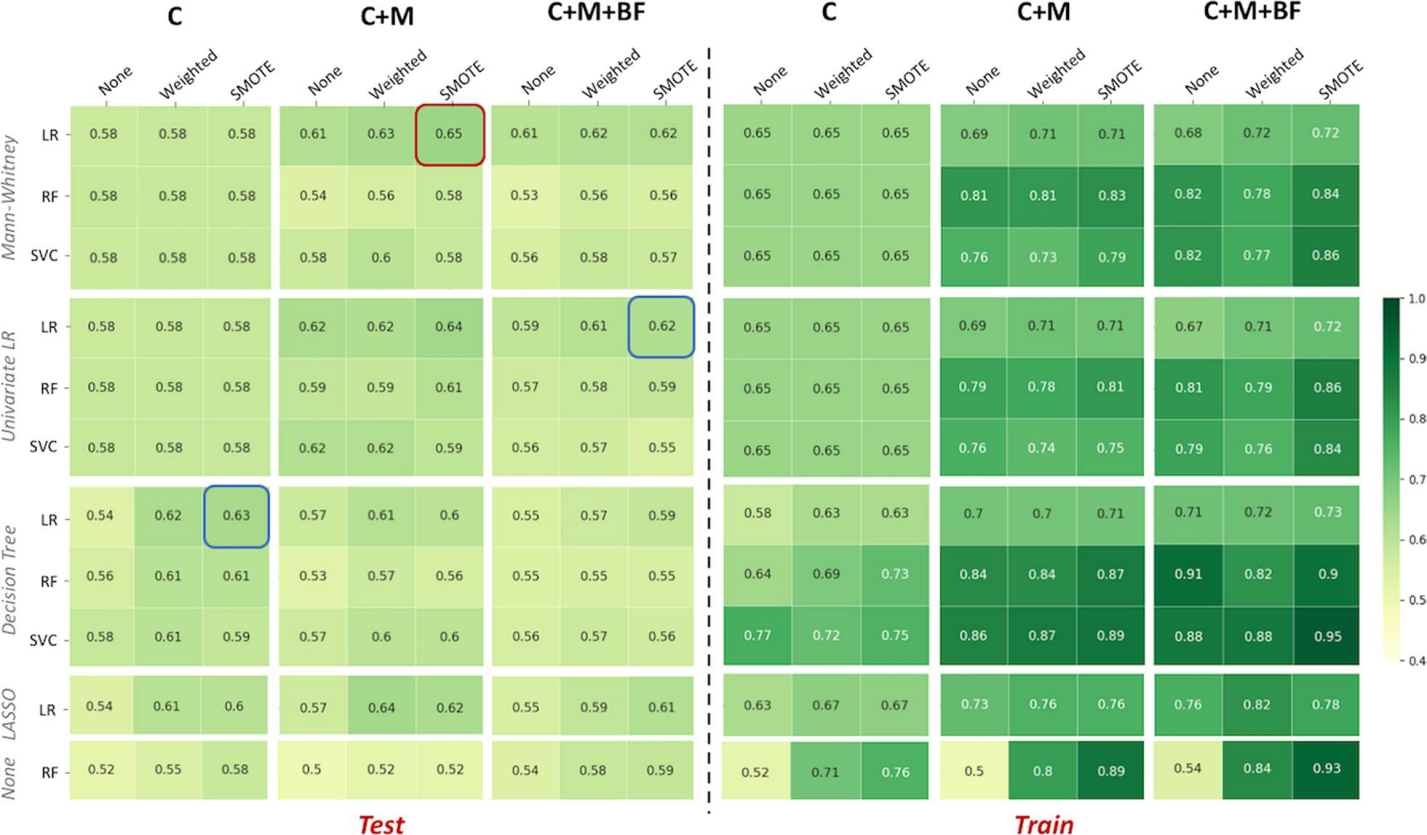
Summary of the balanced accuracy averages over the 50 shuffle splits for the test (n=10) and training (n=42) sets, for each subgroup (C, C+M and C+M+BF) among the TN tumours. The best overall performance is highlighted in red; the other two highest results are highlighted in blue

Considering TN tumours independently, the highest test performance was observed for C+M models, with a mean balanced accuracy higher than C and C+M+BF models (mean BAcc of 0.65 vs. 0.63 and 0.62, respectively), but not significantly (Fig. 6). The best C+M models were noted using a LR classifier combined with a MW feature selection and enhanced by data augmentation using SMOTE.

**Table 6:** Averages over the 50 shuffle splits of the best test and training model performances for each subgroup (C, C+M and C+M+BF) for the TN tumours

	C		C+M		C+M+BF		Dummy Classifier	
	Test	Train	Test	Train	Test	Train	Test	Train
BAcc	0.63	0.63	0.65	0.71	0.62	0.72	0.50	0.50
	$(\pm 0.12)$	$(\pm 0.04)$	$(\pm 0.13)$	$(\pm 0.06)$	$(\pm 0.14)$	$(\pm 0.07)$		
F1-score	0.54	0.59	0.56	0.67	0.51	0.68	0	0
	$(\pm 0.16)$	$(\pm 0.04)$	$(\pm 0.16)$	$(\pm 0.07)$	$(\pm 0.18)$	$(\pm 0.08)$		
AUC	0.63	0.63	0.65	0.71	0.62	0.73	0.50	0.50
	$(\pm 0.12)$	$(\pm 0.04)$	$(\pm 0.13)$	$(\pm 0.06)$	$(\pm 0.14)$	$(\pm 0.07)$		
MCC	0.26	0.25	0.31	0.42	0.26	0.45	0	0
	$(\pm 0.24)$	$(\pm 0.08)$	$(\pm 0.25)$	$(\pm 0.12)$	$(\pm 0.29)$	$(\pm 0.13)$		
Recall	0.60	0.65	0.61	0.73	0.53	0.72	0	0
	$(\pm 0.22)$	$(\pm 0.08)$	$(\pm 0.24)$	$(\pm 0.10)$	$(\pm 0.23)$	$(\pm 0.10)$		
Precision	0.51	0.54	0.56	0.63	0.56	0.65	0	0
	$(\pm 0.16)$	$(\pm 0.04)$	$(\pm 0.19)$	$(\pm 0.07)$	$(\pm 0.24)$	$(\pm 0.07)$		

*BAcc* Balanced Accuracy, *MCC* Matthews Correlation Coefficient, *AUC* Area Under the ROC Curve

The best performance for C models (mean BAcc$=$0.63) was obtained using a decision tree (DT) feature selection and a LR classifier with data enhanced using SMOTE. When BF information was added, the best performances were observed using LR for feature selection and classification, with data augmentation using SMOTE. All the performances of these best models are reported in Table 6. All the models studied presented better classification performances than dummies’ models. The corresponding features selected in more than 50% of the splits are listed in Table 7.

These results were obtained when using a AR for the intensity discretisation method of the PET images, and are consistent with the results obtained using the RR method (supplemental Figure S6). The corresponding results for the best models and the selected features are presented in supplementary Tables S5 and S6.

**Table 7:** Features selected in more than 50% of the shuffle splits of the best models for each subgroup (C, C+M and C+M+BF) when analysing TN tumours

C	C+M	C+M+BF
Age, SBR_Mitose, Menopause	M_energy, M_clustershade, M_correlation, SBR_Mitose	M_entropy, M_correlation, BF_skewness, BF_correlation, SBR_Mitose

*BF* Blood Flow, *M* Metabolism, *SBR* Scarff-Bloom-Richardson

## Discussion

In the present work, when the texture information was extracted after an AR of PET image intensity discretisation, the analysis of the HER2 and TN breast cancer together showed that models using clinical and metabolic data presented the best classification performance for identifying pCR on test datasets. The combination of BF information within the models did not improve their performances which presented lower values for all calculated metrics (Table 2). C+M models were significantly better than models using clinical data only (*p*-value <0.01), unlike C+M+BF models that presented no significant difference in performances with C+M or C models.

To the best of our knowledge, this is the first time that the added value of BF and its heterogeneity for pCR classification at baseline has been analysed and reported in BC literature. Although these C+M+BF models cannot be directly compared with external data, the best models identified in this work, without BF information, can be compared with previous studies focusing on pCR characterisation. Three recent studies [21, 34, 35] have questioned the added value of advanced metabolic texture parameters in addition to clinical data, in the prediction of treatment response at baseline. Considering a large database of 435 patients including all tumour phenotypes (HER2, Luminal, TN), Lee et al. [21] observed better classification performances when clinicopathological information was combined with texture parameters from ^18^F-FDG PET scans, albeit not significantly. They obtained AUCs of 0.64, 0.78 and 0.80 for imaging (PET), clinicopathological and combined models, respectively. In the present study, lower AUCs have been observed (average AUC of $0.66\pm 0.09$), which can be explained by methodological aspects. In the current work, the reported AUC value corresponds to an average over 50 shuffle splits, in contrast to the study of Lee et al. where only one split of the dataset was performed (30% of the population dedicated to the test set). Moreover, the population studied was also different, with the inclusion of Luminal tumours which present a non-pCR prevalence superior to 80%, probably leading to higher classification performances.

In the second study, Lim et al. [35] calculated a radiomic score from PET images which improved the pCR classification. The study was performed on 115 patients, considering all tumour phenotypes, and included a separate cohort of 28 patients for external validation (independent test set (24%)). They obtained AUCs of 0.73 and 0.91 considering the radiomic model alone or combined with clinical data, respectively. The authors however did not compare these performances with models using clinical data only. By considering a radiomic score calculated from texture features instead of using texture features directly in their models, the authors presented an alternative approach that, albeit not straightforwardly comparable with the present results, seems promising for pCR classification.

Finally, Antunovic et al. [34] evaluated the role of advanced imaging features for the prediction of tumour response to NAC by comparing models using clinical and PET statistics with and without the addition of ^18^F-FDG PET texture information. Seventy-nine patients (including HER2, luminal and TN tumours) were analysed. The authors performed internal validation with 100 iterations, rather than isolating an independent test dataset. They reported a maximum average AUC=0.62 obtained using models without PET texture information. Antunovic et al. concluded that the inclusion of second and higher-order imaging PET features did not improve the discriminatory power of the models. This is in contradiction with the present results, where metabolic texture information is selected in the best models in at least more than 50% of the splits (Table 3).

These discrepancies between studies can be extended to the literature [36], where articles have reported significant correlations between PET texture features and pCR [37–39] and others have not [40, 41]. Overall, the recent literature tends to agree that pCR classification at baseline could benefit from image texture information [19, 35, 42–44]. For example, Li et al. [19] reported that a radiomic signature composed of two CT-based texture features, two PET-based texture features and one clinical (age of patients) feature combined outperformed single modality-based predictors for NAC response prediction (AUC=0.73 evaluated on independent test set (30%), pCR: 50%). Montemezzi et al. [42] also evaluated the performance of radiomic features extracted from PET and Dynamic Contrast-Enhanced Magnetic Resonance Imaging (DCE-MRI) through internal validation of their models (leave one out 30 and 60-fold CV). They reported that radiomic features extracted from pre-NAC DCE-MRI and PET/CT scans consistently improved the performances of predictive models when added to clinical, histological and radiological data (maximum validation AUC=0.85, pCR: 33%). Considering either all the tumour phenotypes or only HER2 and TN, radiomics extracted from medical imaging appears promising in the characterisation of pCR to NAC at baseline.

When the analysis focused on the HER2 tumours only, the clinical models showed equivalent classification performance to the C+M models, which were higher than the C+M+BF models. However, the C+M models tended to select more features than the C models (Table 5), which may increase their variability and therefore reduce their robustness compared to the clinical models. No significant difference was found between the classification performance of these models and the C+M+BF models. Considering the relationship between HER2 tumours and angiogenesis [45–48], we might have expected that BF heterogeneity would improve the characterisation of the HER2 tumours and by extension, the prediction of their response to therapy. However, the present results suggest that neither metabolic nor BF information improved model performance at baseline. On the other hand, for TN tumours alone, the C+M models showed the best classification performance, which is in line with the results of the whole population (HER2+TN), and with previous studies that have identified early metabolic response as promising for pCR prediction [49, 50].

TN and HER2 tumours differ in their aggressiveness and outcome, resulting in different treatment options and prognoses. Differences have also been reported at the textural level, with significant differences noted between these two phenotypes in terms of metabolic heterogeneity [23]. In the present results, the classification of HER2 tumours showed lower classification performance than TN tumours, despite the larger number of patients (76 vs. 52 for HER2 and TN respectively). In the present cohort, the HER2 lesions are smaller, less metabolically active and have a lower blood flow than TN tumours (Table 1). Due to these differences, texture analysis of HER2 tumours may suffer from a lack of robustness, especially for blood flow, as small tumour sizes can be a limiting factor for texture analysis, as well as a low signal-to-noise ratio within the image. For HER2 tumours, models using clinical data outperformed the image-based information in predicting pCR, and it is worth noting the high number of clinical features that were selected in more than 50% of the splits for all model configurations (Table 5). For TN tumours, however, all the metabolic PET features selected in the majority of the splits were texture-based only, supporting the added value of texture analysis for pCR prediction in TN tumours.

In all subgroups, the most frequently selected clinical feature was the SBR mitotic class of the tumour. Histopathological grade is known to correlate with response to chemotherapy, with grade III tumours having a better response [51]. The value of the SBR mitotic class appears here as one of the most important predictive clinical factors associated with the therapeutic response. The feature correlation was also selected in most of the models, supporting its contribution to pCR classification. This feature is known to be independent of the intensity discretisation method and robust to the associated number or size of bins used [31].

It should be also noted that for HER2 and HER2 and TN combined, discrepancies have been observed between the results obtained using either a fixed bin size or a fixed number of bins for the intensity discretisation method. Variability in image texture extraction is considered to be one of the major drawbacks in radiomics [52]. Using a RR will quench the minimum and maximum intensities within the lesion. This may be a limitation for noisy lesions with low uptake, where intensity discretisation may only increase the noise in the image. This may be particularly true for our cohort of HER2 lesions, given their characteristics (small volumes, low uptake and perfusion). In this case, it could be questioned whether the contributions of BF features to pCR classification are purely artefactual. However, for TN tumours which had larger tumour volumes and higher uptake and perfusion, the ability of the models to classify patients according to pCR was consistent between the two intensity discretisation methods. In light of these results, an AR for the image intensity discretisation may be preferred for BF PET image analysis.

The present results could be influenced by several limitations. Firstly, as previously discussed, the low count statistics in the dynamic images can induce a bias that may affect the results [53]. By improving the signal-to-noise ratio and the spatial resolution of the dynamic images, enhanced BF images should be obtained, leading to a more accurate characterisation of tumour BF heterogeneity [54]. Tumour perfusion analysis would certainly benefit from new generations of digital PET or dedicated breast PET scanners. Secondly, the small size of the tumours can also be addressed, as it may be a limiting factor for texture analysis. However, small tumour size is one of the characteristics of BC and the extracted texture features were selected taking into account this aspect, by using only local TFs, i.e., computed using GLCM. The discrepancies between studies could also reflect differences in the methodologies of machine learning pipelines, especially in the choice of the validation datasets, which affects the comparability of the results between studies. Furthermore, even when assessing the added value of image texture information in pCR classification, the final texture features selected in the models are usually inconsistent between studies [36]. This highlights the variability of radiomic analyses and their potential lack of robustness. The use of repeated shuffle splits in the present study may reduce the overall model performances obtained compared to a single split, but should guarantee a more robust evaluation of the added value of a specific information (either image-based, clinical, histopathological, etc.). The choice of having an average of the model performances instead of determining a unique radiomic signature was considered here to assess the overall added value of each type of information.

## Conclusion

In this study, we investigated the added value of combining at baseline clinical, histophatological, BF and metabolic features, including image texture information, for pCR prediction in newly diagnosed localised BC. Significantly better classification performance was reported using the combination of metabolic, metabolic texture features and clinical features, for HER2 and TN tumours combined. The addition of BF information to the models did not improve classification performances, regardless of the tumour subgroups analysed.

### Supplementary Information

Supplementary Material 1.

## Data Availability

The datasets used and analysed during the current study are not publicly available but are available from the corresponding author on reasonable request.

## Abbreviations

AUC: Area Under the Curve
BAcc: Balanced Accuracy
BC: Breast Cancer
BF: Blood Flow
C: Clinical
CT: Computed Tomography
CV: Cross Validation
DCE-MRI: Dynamic Contrast-Enhanced Magnetic Resonance Imaging
DT: Decision Trees
ER: Estrogen Receptor
FDG: Fluorodeoxyglucose
FISH: Fluorescent In Situ Hybridisation
GLCM: Gray Level Co-occurrence Matrix
IBSI: Image Biomarker Standardisation Initiative
IDM: Inverse Different Moment
ITK: Insight Segmentation and Registration Toolkit
LASSO: Least Absolute Shrinkage and Selection Operator
LR: Logistic Regression
M: Metabolism
MATV: Metabolic Active Tumour Volume
MCC: Matthews Correlation Coefficient
NAC: Neoadjuvant Chemotherapy
pCR: Pathological Complete Response
PET: Positron Emission Tomography
PR: Progesterone Receptor
RF: Random Forest
SBR: Scarff-Bloom-Richardson
SMOTE: Syntactic Minority Over-sampling Technique
SUV: Standardized Uptake Value
SVC: Support Vector Classification
TA: Texture Analysis
TLG: Tumour Lesion Glycolysis
TN: Triple Negative
VOI: Volume Of Interest

## References

[1] Wilkinson L, Gathani T (2021). Understanding breast cancer as a global health concern. Br J Radiol..

[2] Li N, Deng Y, Zhou L, Tian T, Yang S, Wu Y (2019). Global burden of breast cancer and attributable risk factors in 195 countries and territories, from 1990 to 2017: results from the Global Burden of Disease Study 2017. J Hematol Oncol..

[3] Sharma R (2021). Global, regional, national burden of breast cancer in 185 countries: evidence from GLOBOCAN 2018. Breast Cancer Res Treat..

[4] Perou CM, Sørlie T, Eisen MB, van de Rijn M, Jeffrey SS, Rees CA (2000). Molecular portraits of human breast tumours. Nature..

[5] Zardavas D, Irrthum A, Swanton C, Piccart M (2015). Clinical management of breast cancer heterogeneity. Nat Rev Clin Oncol..

[6] Kong X, Moran MS, Zhang N, Haffty B, Yang Q (2011). Meta-analysis confirms achieving pathological complete response after neoadjuvant chemotherapy predicts favourable prognosis for breast cancer patients. Eur J Cancer..

[7] Cortazar P, Zhang L, Untch M, Mehta K, Costantino JP, Wolmark N (2014). Pathological complete response and long-term clinical benefit in breast cancer: the CTNeoBC pooled analysis. Lancet..

[8] Hurtz HJ, Tesch H, Göhler T, Hutzschenreuter U, Harde J, Kruggel L (2017). Persistent impairments 3 years after (neo)adjuvant chemotherapy for breast cancer: results from the MaTox project. Breast Cancer Res Treat..

[9] Han S, Choi JY (2020). Prognostic value of F-FDG PET and PET/CT for assessment of treatment response to neoadjuvant chemotherapy in breast cancer: a systematic review and meta-analysis. Breast Cancer Res..

[10] Champion L, Lerebours F, Alberini JL, Fourme E, Gontier E, Bertrand F (2015). 18F-FDG PET/CT to Predict Response to Neoadjuvant Chemotherapy and Prognosis in Inflammatory Breast Cancer. J Nucl Med..

[11] Lee SM, Bae SK, Kim TH, Yoon HK, Jung SJ, Park JS (2014). Value of 18F-FDG PET/CT for early prediction of pathologic response (by residual cancer burden criteria) of locally advanced breast cancer to neoadjuvant chemotherapy. Clin Nucl Med..

[12] Cochet A, Pigeonnat S, Khoury B, Vrigneaud JM, Touzery C, Berriolo-Riedinger A (2012). Evaluation of breast tumor blood flow with dynamic first-pass 18F-FDG PET/CT: comparison with angiogenesis markers and prognostic factors. J Nucl Med..

[13] Dunnwald LK, Gralow JR, Ellis GK, Livingston RB, Linden HM, Specht JM (2008). Tumor metabolism and blood flow changes by positron emission tomography: relation to survival in patients treated with neoadjuvant chemotherapy for locally advanced breast cancer. J Clin Oncol..

[14] Humbert O, Riedinger JM, Vrigneaud JM, Kanoun S, Dygai-Cochet I, Berriolo-Riedinger A (2016). 18F-FDG PET-Derived Tumor Blood Flow Changes After 1 Cycle of Neoadjuvant Chemotherapy Predicts Outcome in Triple-Negative Breast Cancer. J Nucl Med..

[15] Mankoff DA, Dunnwald LK, Partridge SC, Specht JM (2009). Blood flow-metabolism mismatch: good for the tumor, bad for the patient. Clin Cancer Res..

[16] Hatt M, Tixier F, Pierce L, Kinahan PE, Le Rest CC, Visvikis D (2017). Characterization of PET/CT images using texture analysis: the past, the present any future?. Eur J Nucl Med Mol Imaging..

[17] Gillies RJ, Kinahan PE, Hricak H (2016). Radiomics: Images Are More than Pictures. They Are Data. Radiology..

[18] Marusyk A, Janiszewska M, Polyak K (2020). Intratumor heterogeneity: the rosetta stone of therapy resistance. Cancer Cell..

[19] Li P, Wang X, Xu C, Liu C, Zheng C, Fulham MJ (2020). F-FDG PET/CT radiomic predictors of pathologic complete response (pCR) to neoadjuvant chemotherapy in breast cancer patients. Eur J Nucl Med Mol Imaging..

[20] Roy S, Whitehead TD, Li S, Ademuyiwa FO, Wahl RL, Dehdashti F (2022). Co-clinical FDG-PET radiomic signature in predicting response to neoadjuvant chemotherapy in triple-negative breast cancer. Eur J Nucl Med Mol Imaging..

[21] Lee H, Lee DE, Park S, Kim TS, Jung SY, Lee S (2019). Predicting Response to Neoadjuvant Chemotherapy in Patients With Breast Cancer: Combined Statistical Modeling Using Clinicopathological Factors and FDG PET/CT Texture Parameters. Clin Nucl Med..

[22] Groheux D, Giacchetti S, Moretti JL, Porcher R, Espié M, Lehmann-Che J (2011). Correlation of high 18F-FDG uptake to clinical, pathological and biological prognostic factors in breast cancer. Eur J Nucl Med Mol Imaging..

[23] Payan N, Presles B, Brunotte F, Coutant C, Desmoulins I, Vrigneaud JM (2020). Biological correlates of tumor perfusion and its heterogeneity in newly diagnosed breast cancer using dynamic first-pass F-FDG PET/CT. Eur J Nucl Med Mol Imaging..

[24] Amin MB, Edge SB, Greene FL, Byrd DR, Brookland RK, Washington MK (2018). AJCC Cancer Staging Manual.

[25] Chevallier B, Roche H, Olivier JP, Chollet P, Hurteloup P (1993). Inflammatory breast cancer. Pilot study of intensive induction chemotherapy (FEC-HD) results in a high histologic response rate. Am J Clin Oncol..

[26] Leijenaar RTH, Nalbantov G, Carvalho S, van Elmpt WJC, Troost EGC, Boellaard R (2015). The effect of SUV discretization in quantitative FDG-PET Radiomics: the need for standardized methodology in tumor texture analysis. Sci Rep..

[27] Zwanenburg A, Leger S, Vallières M, Löck S. Image biomarker standardisation initiative. Website. Accessed: 10.48550/arXiv.1612.07003.

[28] Orlhac F, Nioche C, Soussan M, Buvat I (2017). Understanding changes in tumor texture indices in PET: a comparison between visual assessment and index values in simulated and patient data. J Nucl Med..

[29] Hatt M, Majdoub M, Vallières M, Tixier F, Le Rest CC, Groheux D (2015). 18F-FDG PET uptake characterization through texture analysis: investigating the complementary nature of heterogeneity and functional tumor volume in a multi-cancer site patient cohort. J Nucl Med..

[30] Shen WC, Chen SW, Liang JA, Hsieh TC, Yen KY, Kao CH (2017). [18] Fluorodeoxyglucose positron emission tomography for the textural features of cervical cancer associated with lymph node metastasis and histological type. Eur J Nucl Med Mol Imag..

[31] Payan N, Presles B, Truntzer C, Courcet E, Coutant C, Desmoulins I (2022). Critical analysis of the effect of various methodologies to compute breast cancer tumour blood flow-based texture features using first-pass F-FDG PET. Phys Med..

[32] McCormick M, Liu X, Jomier J, Marion C, Ibanez L (2014). ITK: enabling reproducible research and open science. Front Neuroinform..

[33] Schaefer A, Kremp S, Hellwig D, Rübe C, Kirsch CM, Nestle U (2008). A contrast-oriented algorithm for FDG-PET-based delineation of tumour volumes for the radiotherapy of lung cancer: derivation from phantom measurements and validation in patient data. Eur J Nucl Med Mol Imaging..

[34] Antunovic L, De Sanctis R, Cozzi L, Kirienko M, Sagona A, Torrisi R (2019). PET/CT radiomics in breast cancer: promising tool for prediction of pathological response to neoadjuvant chemotherapy. Eur J Nucl Med Mol Imaging..

[35] Lim CH, Choi JY, Choi JH, Lee JH, Lee J, Lim CW (2023). Development and External Validation of F-FDG PET-Based Radiomic Model for Predicting Pathologic Complete Response after Neoadjuvant Chemotherapy in Breast Cancer. Cancers..

[36] Oliveira C, Oliveira F, Vaz SC, Marques HP, Cardoso F (2023). Prediction of pathological response after neoadjuvant chemotherapy using baseline FDG PET heterogeneity features in breast cancer. Br J Radiol..

[37] Molina-García D, García-Vicente AM, Pérez-Beteta J, Amo-Salas M, Martínez-González A, Tello-Galán MJ (2018). Intratumoral heterogeneity in F-FDG PET/CT by textural analysis in breast cancer as a predictive and prognostic subrogate. Ann Nucl Med..

[38] Yoon HJ, Kim Y, Chung J, Kim BS (2019). Predicting neo-adjuvant chemotherapy response and progression-free survival of locally advanced breast cancer using textural features of intratumoral heterogeneity on F-18 FDG PET/CT and diffusion-weighted MR imaging. Breast J..

[39] Ha S, Park S, Bang JI, Kim EK, Lee HY (2017). Metabolic Radiomics for Pretreatment F-FDG PET/CT to Characterize Locally Advanced Breast Cancer: Histopathologic Characteristics, Response to Neoadjuvant Chemotherapy, and Prognosis. Sci Rep..

[40] Lemarignier C, Martineau A, Teixeira L, Vercellino L, Espié M, Merlet P (2017). Correlation between tumour characteristics, SUV measurements, metabolic tumour volume, TLG and textural features assessed with F-FDG PET in a large cohort of oestrogen receptor-positive breast cancer patients. Eur J Nucl Med Mol Imaging..

[41] Cheng L, Zhang J, Wang Y, Xu X, Zhang Y, Zhang Y (2017). Textural features of F-FDG PET after two cycles of neoadjuvant chemotherapy can predict pCR in patients with locally advanced breast cancer. Ann Nucl Med..

[42] Montemezzi S, Benetti G, Bisighin MV, Camera L, Zerbato C, Caumo F (2021). 3T DCE-MRI Radiomics Improves Predictive Models of Complete Response to Neoadjuvant Chemotherapy in Breast Cancer. Front Oncol..

[43] Yang L, Chang J, He X, Peng M, Zhang Y, Wu T (2022). PET/CT-based radiomics analysis may help to predict neoadjuvant chemotherapy outcomes in breast cancer. Front Oncol..

[44] Umutlu L, Kirchner J, Bruckmann NM, Morawitz J, Antoch G, Ting S (2022). Multiparametric F-FDG PET/MRI-based radiomics for prediction of pathological complete response to neoadjuvant chemotherapy in breast cancer. Cancers..

[45] Konecny GE, Meng YG, Untch M, Wang HJ, Bauerfeind I, Epstein M (2004). Association between HER-2/neu and vascular endothelial growth factor expression predicts clinical outcome in primary breast cancer patients. Clin Cancer Res..

[46] Kumar R, Yarmand-Bagheri R (2001). The role of HER2 in angiogenesis. Semin Oncol..

[47] Yang W, Klos K, Yang Y, Smith TL, Shi D, Yu D (2002). ErbB2 overexpression correlates with increased expression of vascular endothelial growth factors A, C, and D in human breast carcinoma. Cancer..

[48] Ahlgren J, Risberg B, Villman K, Bergh J (2002). Angiogenesis in invasive breast carcinoma-a prospective study of tumour heterogeneity. Eur J Cancer..

[49] Humbert O, Riedinger JM, Charon-Barra C, Berriolo-Riedinger A, Desmoulins I, Lorgis V (2015). Identification of Biomarkers Including 18FDG-PET/CT for Early Prediction of Response to Neoadjuvant Chemotherapy in Triple-Negative Breast Cancer. Clin Cancer Res..

[50] Groheux D, Biard L, Giacchetti S, Teixeira L, Hindié E, Cuvier C (2016). 18F-FDG PET/CT for the early evaluation of response to neoadjuvant treatment in triple-negative breast cancer: influence of the chemotherapy regimen. J Nucl Med..

[51] Pinder SE, Murray S, Ellis IO, Trihia H, Elston CW, Gelber RD, et al. The importance of the histologic grade of invasive breast carcinoma and response to chemotherapy. Cancer. 1998 10;83(8):1529–1539. 10.1002/(SICI)1097-0142(19981015)83:8<1529::AID-CNCR7>3.0.CO;2-V. 9781946

[52] Pesapane F, Rotili A, Agazzi GM, Botta F, Raimondi S, Penco S (2021). Recent Radiomics Advancements in Breast Cancer: Lessons and Pitfalls for the Next Future. Curr Oncol..

[53] Presotto L, Bettinardi V, De Bernardi E, Belli ML, Cattaneo GM, Broggi S (2018). PET textural features stability and pattern discrimination power for radiomics analysis: An “ad-hoc” phantoms study. Phys Med..

[54] Moscoso A, Ruibal Á, Domínguez-Prado I, Fernández-Ferreiro A, Herranz M, Albaina L (2018). Texture analysis of high-resolution dedicated breast F-FDG PET images correlates with immunohistochemical factors and subtype of breast cancer. Eur J Nucl Med Mol Imaging..

