# Supplementary material for: Respective contribution of baseline clinical data, tumour metabolism and tumour blood-flow in predicting pCR after neoadjuvant chemotherapy in HER2 and Triple Negative breast cancer
Source: EJNMMI Res. 2024 Jul 4;14:60. doi: 10.1186/s13550-024-01115-4 (PMC11224181; doi:10.1186/s13550-024-01115-4)
Supplement: Supplementary file 1 — Supplementary Material 1. [file 13550_2024_1115_MOESM1_ESM.pdf]

Supplementary Material

Neree Payan, Benoit Presles, Charles Coutant,
Isabelle Desmoulins, Sylvain Ladoire, Françoise Beltjens,
François Brunotte, Jean-Marc Vrigneaud, Alexandre Cochet

1 Blood flow and Metabolism analyses

The tumour perfusion measurements were assessed using dynamic PET of first pass ^{18}F-FDG uptake based on the one-compartment first-pass kinetic model of Mullani et al. [1]. Volumes of interest (VOIs) were manually delineated on the early 8-min PET image with the help of the corresponding anatomical image using a dedicated workstation (Extended Brilliance Workspace 3.5; Phillips): one volume representing the contours of the perfused primary tumour, one VOI centred on the ascending aorta and a third VOI delineated within the contralateral normal breast. All these volumes were superimposed on the dynamic PET image. Using in-house scripts, parametric blood-flow images, containing a BF value in each tumour voxel, were generated using the BF equation reported in [2, 3]. The tumour metabolism analyses were performed by first delineating Metabolic Active Tumour Volumes (MATV) on the delayed PET images (90-min post injection) using a semi-automatic segmentation algorithm based on a contrast-dependent method [4] as explained in [2]. The delayed PET images were then converted into SUV.

2 PET-derived data

The calculated features were the intensity average (BFmean and SUVmean), standard deviation (BFsd and SUVsd), and maximum (BFmax and SUVmax) tumour values. Skewness and kurtosis histogram intensity-based parameters were also computed. The MATV and the total lesion glycolysis (TLG) were extracted from the SUV images. Tumour perfused volumes were extracted from BF images. In addition to these intensity (7), intensity histogram (4) and shape (2) features, 18 TFs based on the GLCM were calculated. These TFs were: the homogeneity, the inverse different moment (IDM), the energy, the entropy, the contrast, the dissimilarity, the correlation, the cluster shade and the cluster prominence extracted from both, perfusion and metabolic parametric images.

3 Clinical and histopathological data

The age of the patients was reported as well as their menopausal status (post-menopausal or not) and whether the tumours were pluri-focal or not. From core biopsies, histological type and tumour grade, using the Scarff-Bloom-Richardson modified by Elston and Ellis (SBR) system, were included. The SBR grade and mitotic SBR class were classified as high grade (grade 3) vs. low grade (grade 1 and 2). From patients' TNM grading, the tumour (T1 and T2 vs T3 and T4) and lymph node (N0 vs N1-3) status were reported. The hormone receptor status (PR and ER) and the type of treatment were also included in the data.

4 Machine learning pipelines

4.1 Features Selection

Three types of classification algorithms have been tested: Logistic Regression (LR), Support Vector Classification (SVC) and Random Forest (RF) classifiers, each associated with several feature selection methods. Univariate feature selections using two-sided Mann-Whitney tests or univariate LR were implemented, considering several p-values as significance criteria: 0.10, 0.15 and 0.20. A wrapper feature selection using Decision Trees (DT) was also carried out. In addition, an embedded feature selection method was implemented, using the least absolute shrinkage and selection operator (LASSO) penalisation, when associated with the LR classifier. Finally, since RF classifiers are known to be robust against correlated features, no feature selection was investigated in addition to the above-mentioned feature selection methods, when associated with this classification algorithm. Spearman correlations between all the features were also calculated either before the wrapper and embedded feature selections or after the univariate feature selection since the latter considers the variables independently. To reduce multicollinearity, all the highly correlated features were removed using a cut off value of $\rho > 0.8$. To prevent any bias, all the feature selections were performed using data from the training cohort only.

4.2 Models' developments

Models' hyperparameters were optimised via a grid-search on the training dataset, using an inner 4-fold cross validation (CV). A decision threshold of 0.5 was used for the hard classification performance metrics. Class balancing was investigated using weighted classes or by applying data augmentation using the Syntactic Minority Over-sampling Technique (SMOTE) with over-samplings. All the optimal models were selected using a balanced accuracy.

References

[1] Mullani NA, Herbst RS, O'Neil RG, Gould KL, Barron BJ, Abbruzzese JL. Tumor blood flow measured by PET dynamic imaging of first-pass 18F-FDG uptake: a comparison with 15O-labeled water-measured blood flow. J Nucl Med. 2008 Apr;49(4):517–523.

[2] Payan N, Presles B, Brunotte F, Coutant C, Desmoulins I, Vrigneaud JM, et al. Biological correlates of tumor perfusion and its heterogeneity in newly diagnosed breast cancer using dynamic first-pass F-FDG PET/CT. Eur J Nucl Med Mol Imaging. 2020 May;47(5):1103–1115.

[3] Cochet A, Pigeonnat S, Khoury B, Vrigneaud JM, Touzery C, Berriolo-Riedinger A, et al. Evaluation of breast tumor blood flow with dynamic first-pass 18F-FDG PET/CT: comparison with angiogenesis markers and prognostic factors. J Nucl Med. 2012 Apr;53(4):512–520.

[4] Schaefer A, Kremp S, Hellwig D, Rübe C, Kirsch CM, Nestle U. A contrast-oriented algorithm for FDG-PET-based delineation of tumour volumes for the radiotherapy of lung cancer: derivation from phantom measurements and validation in patient data. Eur J Nucl Med Mol Imaging. 2008 Nov;35(11):1989–1999.

IBSI biomarkers computation	
**Intensity Discretisation method**	Fixed bin size: 0.016 mL/min/g for blood flow and 0.47 SUV for metabolism, with the lowest intensity in the first bin equal to 0 mL/min/g and 0 SUV.<br>Fixed number of bins: 64
**Biomarker set**	**Clinical and histopathological**: Age, phenotype, estrogene receptor, progesterone receptor, menopause, pluri-focal, N stage, T stage, SBR, SBR mitotic, treatment type<br>**Intensity-based statistical PET-derived features**: SUV mean, SUV max, SUV standard deviation (sd), Metabolic active tumour volume (MATV), Total lesion glycolysis (TLG), BF mean, BF max, BF sd, Perfused volume, Intensity skewness (BF and metabolic), Intensity kurtosis (BF and metabolic)<br>**GLCM features:** Energy, Entropy, Homogeneity, Inverse different moment (IDM), Contrast, Correlation, Dissimilarity, Cluster shade, Cluster prominence (BF and metabolic)
**IBSI compliance**	In-house tools based on the Insight Segmentation and Registration Toolkit (ITK) library (IBSI compliant)
**Software availability**	https://github.com/benpresles/vv
**Texture matrix aggregation**	13 GLCM (one for each spatial direction)
**CM symmetry**	Symmetric
**CM distance**	Chebyshev distance of 1

Fig. S1 IBSI: PET image processing and biomarkers computation

Protocol and data descriptions	
**Volume of interest**	Metabolic Active Tumour Volume (MATV) and Perfused Volume (PV)
**Patient preparation**	Patients were required to fast for at least 6h before injection of 3 MBq/kg of ^{18}F-FDG
**Radiotracer**	[^{18}F]-FDG / 2-Deoxy-2-[18F]-fluoroglucose<br>Injection of a bolus of 3 MBq/kg with an automatic PET infusion system (Bayer Medical Care, Inc., Indianola, PA, USA).
**Acquisition protocol**	Simultaneously to the injection, a first 8-min list-mode F-FDG PET scan is acquired in a prone position, centred on the breast. Whole-body emission and transmission scans were acquired 60 min later in a supine position, followed by a delayed two-step PET/CT scan restricted to the chest, acquired in a prone position 90 min after injection. The latter was performed with 4 min per bed position.
**Scanner type**	Gemini TruFlight PET/CT scanner (Philips Medical Systems, Eindhoven, The Netherlands)
**PET Static/dynamic scans**	Two series of images were used:<br>- A dynamic first-pass image was reconstructed based on the twelve 10-s frames extracted from the first two minutes of the early static PET acquisition.<br>- A delayed two-step PET/CT scan restricted to the chest, acquired in a prone position 90 min after injection with 4 min per bed position.
**^{18}F-FDG PET**	Voxel size: 4x4x4 mm^3<br>Matrix size: 144x144<br>Slice thickness: 4 mm<br>Image slice spacing: 0 mm<br>Reconstruction: 3D OSEM (three iterations and 33 subsets) TOF<br>Corrections: random coincidences, decay, dead time, scatter and attenuation
**CT**	Tube voltage: 120 kVp<br>Tube current: automatic current modulation<br>0.5-s rotation time<br>16 x 1.5 mm collimation<br>pitch of 0.7
**Blood flow data**	Computed using one-compartment first-pass kinetic model of Mullani et al.
**Segmentation method**	Reference image: PET<br>Perfused volume: manual delineation performed by 2 experts<br>MATV: semi-automatic segmentation algorithm based on a contrast-dependent method (Schaefer A et al. 2008) as explained in Payan et al. 2020.

Fig. S2 Protocol and PET/CT data description

Machine learning parameters	
**Spearman correlation**	Cut off value of ρ > 0.8
**Mann Whitney feature selection**	Mannwhitneyu() from scipy- stats library<br>alternative=two-sided<br>p-values as significance criteria: 0.10, 0.15 and 0.20.
**Univariate logistic regression feature selection**	LogisticRegression() function from sklearn - linear_model library<br>penalty=none<br>max_iter= 20000<br>fit_intercept=False<br>solver=saga<br>random_state=42<br>p-values as significance criteria: 0.10, 0.15 and 0.20.
**Decision Tree feature selection**	ExtraTreesClassifier() function from sklearn – ensemble library<br>n_estimators=100<br>max_features=None,<br>min_samples_leaf = [1, 2, 4, 6]<br>random_state=42<br>cv=10
**LASSO feature selection and classification**	LogisticRegression() function from sklearn - linear_model library<br>penalty=l1<br>max_iter= 20000000<br>fit_intercept=True<br>solver=liblinear<br>random_state=42<br>C_list = [0.1, 0.2, 0.5, 0.8, 1, 3, 5, 8, 10, 15, 100] for HER2 + TN<br>C_list = [0.5, 0.8, 1, 3, 5, 8, 10, 15, 100] for HER2 and TN separatly
**Logistic regression classifier**	LogisticRegression() function from sklearn - linear_model library<br>penalty=l2<br>max_iter= 20000000<br>fit_intercept=True<br>solver=lbfgs<br>random_state=42<br>param_grid= [0.001, 0.01, 0.1, 1, 10, 100, 1000, 10000, 1e9]
**SVC classifier**	SVC() function from sklearn library<br>random_state=42<br>param_grid= {'kernel': ['rbf'], 'gamma': [0.0001, 0.001, 0.01, 0.1, 1],<br>'C': [0.0001, 0.001, 0.01, 0.1, 1, 10, 100, 1000, 10000, 1e9]}
**Random Forest classifier**	RandomForestClassifier() function from sklearn – ensemble library<br>param_grid= {'min_samples_leaf': [2, 3, 4, 5, 6, 8, 10, 12, 14, 20, 24]}<br>random_state=42
**Data augmentation**	SMOTE() function from imblearn library<br>sampling_strategy = 'minority'<br>random_state=42

Fig. S3 Machine learning algorithms parameters

5 Results using a fixed number of bins (RR)

5.1 HER2 and TN

The highest performances were noted for C+M+BF models (mean BAcc=0.64) using a LR classifier with LASSO feature selection, enhanced by data augmentation using SMOTE. Close performances were observed for C+M models, with the highest mean balanced accuracy equal to 0.63 when using LR classifier with LASSO feature selection, on weighted data, as reported in Figure S4.
All the performances of these best models are reported in Table S1. All the models studied presented better classification performances than dummies' models. The corresponding features selected in more than 50% of the splits are listed in Table S2.

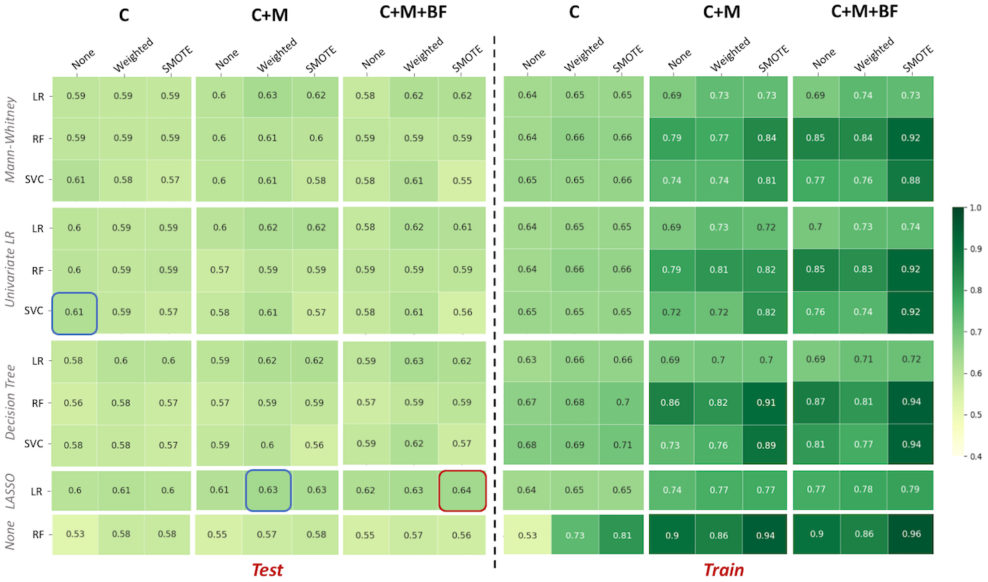

Fig. S4 Summary of the mean balanced accuracy over the 50 shuffle splits for the test (n=26) and training (n=102) sets, for each subgroup (C, C+M and C+M+BF) among the HER2 and TN tumours combined. The best overall performance is highlighted in red; the other two highest results are highlighted in blue.

Table S1 Averages over the 50 shuffle splits of the best test and training model performances for each subgroup (C, C+M and C+M+BF) for the HER2 and TN tumours combined.

	C		C+M		C+M+BF		Dummy Classifier	
	Test	Train	Test	Train	Test	Train	Test	Train
BAcc	0.61 (±0.08)	0.65 (±0.03)	0.63 (±0.09)	0.77 (±0.04)	0.64 (±0.09)	0.79 (±0.04)	0.50	0.50
F1-score	0.49 (±0.14)	0.54 (±0.09)	0.55 (±0.12)	0.71 (±0.05)	0.55 (±0.13)	0.74 (±0.05)	0	0
AUC	0.61 (±0.08)	0.65 (±0.03)	0.63 (±0.09)	0.77 (±0.04)	0.64 (±0.09)	0.79 (±0.04)	0.50	0.50
MCC	0.24 (±0.16)	0.30 (±0.06)	0.27 (±0.19)	0.52 (±0.08)	0.28 (±0.18)	0.57 (±0.09)	0	0
Recall	0.45 (±0.15)	0.51 (±0.10)	0.57 (±0.16)	0.76 (±0.06)	0.58 (±0.18)	0.79 (±0.05)	0	0
Precision	0.55 (±0.15)	0.57 (±0.09)	0.54 (±0.12)	0.66 (±0.05)	0.55 (±0.10)	0.70 (±0.06)	0	0

BAcc: Balanced Accuracy, MCC: Matthews Correlation Coefficient, AUC: Area Under the ROC Curve

Table S2 Features selected in more than 50% of the shuffle splits of the best models for each subgroup (C, C+M and C+M+BF) when analysing HER2 and TN tumours combined.

C	C+M	C+M+BF
SBR, SBR_Mitose, ER	M_sd, M_kurtosis, TLG, M_contrast, M_clustershade, M_clusterprominence, Age, N_Stage, T_Stage, SBR, SBR_Mitose, Menopause, Pluri-focal, Phenotype, ER, RP, Treatment	M_sd, M_kurtosis, TLG, M_contrast, M_clustershade, M_clusterprominence, BF_mean, BF_kurtosis, BF_IDM, BF_clustershade, BF_clusterprominence, Age, N_Stage, T_Stage, SBR, SBR_Mitose, Menopause, Pluri-focal, Phenotype, ER, RP, Treatment

BF: Blood Flow, ER: Estrogen Receptor M: Metabolism, PR: Progesteron Receptor, SBR: Scarff-Bloom-Richardson, TLG: Total Lesion Glycolysis

5.2 HER2

Considering HER2 tumours independently, the highest test performances were observed for C+M+BF models, with a mean balanced accuracy of 0.66. Clinical and C+M models presented equal performances for their best models with a mean balanced accuracy equal to 0.64. All these models used a LR classifier with LASSO feature selection, on weighted data as shown in Figure S5.
The performances of these best models are reported in Table S3. All the models studied presented better classification performances than dummies' models. The corresponding features selected in more than 50% of the splits are listed in Table S4.

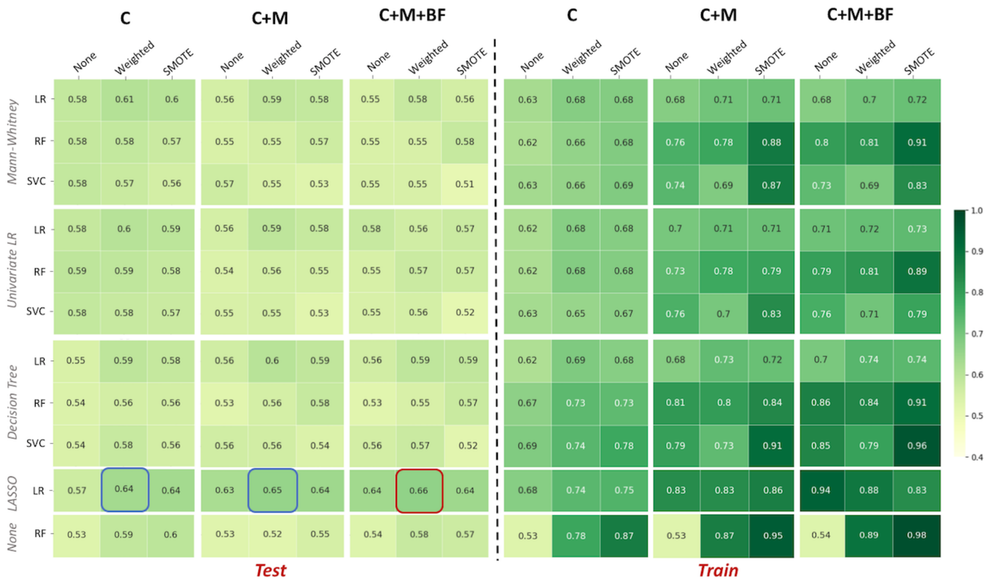

Fig. S5 Summary of the mean balanced accuracy over the 50 shuffle splits for the test (n=15) and training (n=61) sets, for each subgroup (C, C+M and C+M+BF) among the HER2 tumours. The best overall performance is highlighted in red; the other two highest results are highlighted in blue.

Table S3 Averages over the 50 shuffle splits of the best test and training model performances for each subgroup (C, C+M and C+M+BF) for the HER2 tumours.

	C		C+M		C+M+BF		Dummy Classifier	
	Test	Train	Test	Train	Test	Train	Test	Train
BAcc	0.64 (±0.12)	0.74 (±0.04)	0.65 (±0.11)	0.83 (±0.06)	0.66 (±0.12)	0.88 (±0.06)	0.50	0.50
F1-score	0.55 (±0.16)	0.66 (±0.04)	0.56 (±0.15)	0.77 (±0.07)	0.57 (±0.15)	0.84 (±0.07)	0	0
AUC	0.64 (±0.12)	0.74 (±0.04)	0.65 (±0.11)	0.83 (±0.06)	0.66 (±0.12)	0.88 (±0.06)	0.50	0.50
MCC	0.29 (±0.24)	0.46 (±0.07)	0.32 (±0.23)	0.64 (±0.12)	0.33 (±0.23)	0.74 (±0.12)	0	0
Recall	0.58 (±0.20)	0.73 (±0.07)	0.58 (±0.19)	0.85 (±0.06)	0.57 (±0.20)	0.90 (±0.07)	0	0
Precision	0.56 (±0.16)	0.61 (±0.04)	0.59 (±0.17)	0.71 (±0.08)	0.60 (±0.17)	0.78 (±0.09)	0	0

BAcc: Balanced Accuracy, MCC: Matthews Correlation Coefficient, AUC: Area Under the ROC Curve

5.3 TN

When only TN tumours were analysed, the highest performances (mean Bacc=0.65) were noted for C+M models using LR classifier combined with a MW feature selection, and enhanced by data augmentation using SMOTE. When BF information was

Table S4 Features selected in more than 50% of the shuffle splits of the best models for each subgroup (C, C+M and C+M+BF) when analysing HER2 tumours.

C	C+M	C+M+BF
N_Stage, T_Stage, SBR, SBR_Mitose, Menopause, Pluri-focal, ER, Treatment	M_sd, M_kurtosis,M_IDM, M_skewness, M_contrast, M_clustershade, M_clusterprominence, TLG, Age, N_Stage, T_Stage, SBR, SBR_Mitose, Menopause, Pluri-focal, ER, PR, Treatment	M_sd, M_contrast, M_clustershade, BF_mean, BF_clusterprominence, N_Stage, T_Stage, SBR, SBR_Mitose, Pluri-focal, ER, PR, Treatment

BF: Blood Flow, ER: Estrogen Receptor, IDM: Inverse Different Moment, M: Metabolism, PR: Progesteron Receptor, SBR: Scarff-Bloom-Richardson, TLG: Total Lesion Glycolysis

added, the best performances were noted using a DT feature selection combined with a LR classifier, on weighted data (mean BAcc=0.62) (Fig S6).
All the performances of these best models are reported in Table S5. All the models studied presented better classification performances than dummies' models. The corresponding features selected in more than 50% of the splits are listed in Table S6.

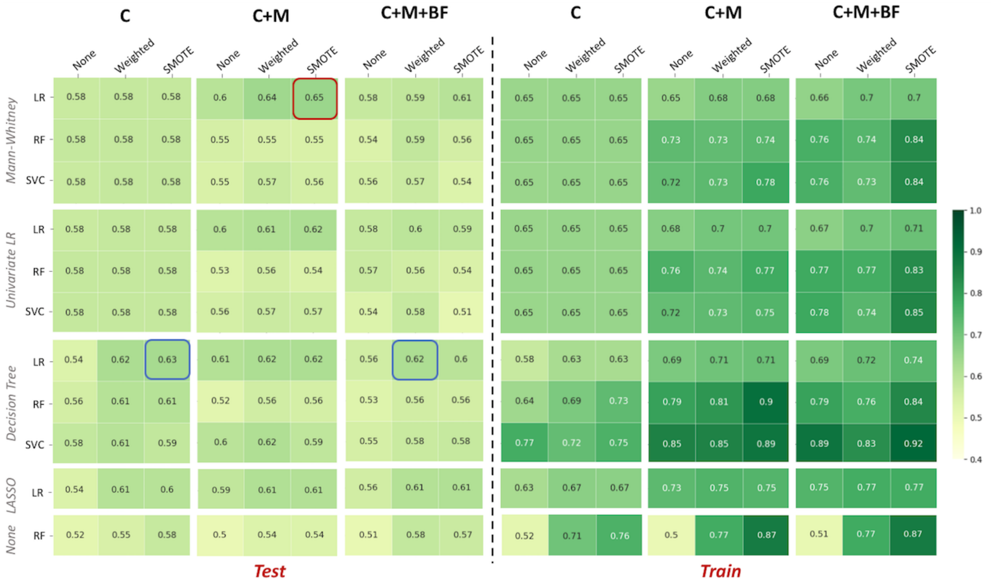

Fig. S6 Summary of the mean balanced accuracy over the 50 shuffle splits for the test (n=10) and training (n=42) sets, for each subgroup (C, C+M and C+M+BF) among the TN tumours. The best overall performance is highlighted in red; the other two highest results are highlighted in blue.

Table S5 Averages over the 50 shuffle splits of the best test and training model performances for each subgroup (C, C+M and C+M+BF) for the TN tumours.

	C		C+M		C+M+BF		Dummy Classifier	
	Test	Train	Test	Train	Test	Train	Test	Train
BAcc	0.63 (±0.12)	0.63 (±0.04)	0.65 (±0.13)	0.68 (±0.04)	0.62 (±0.13)	0.72 (±0.05)	0.50	0.50
F1-score	0.54 (±0.16)	0.59 (±0.04)	0.53 (±0.19)	0.64 (±0.05)	0.51 (±0.20)	0.68 (±0.06)	0	0
AUC	0.63 (±0.12)	0.63 (±0.04)	0.65 (±0.13)	0.68 (±0.04)	0.62 (±0.13)	0.72 (±0.05)	0.50	0.50
MCC	0.26 (±0.24)	0.25 (±0.08)	0.30 (±0.26)	0.36 (±0.09)	0.24 (±0.27)	0.43 (±0.10)	0	0
Recall	0.60 (±0.22)	0.65 (±0.08)	0.57 (±0.26)	0.68 (±0.09)	0.56 (±0.25)	0.73 (±0.07)	0	0
Precision	0.51 (±0.16)	0.54 (±0.04)	0.54 (±0.18)	0.61 (±0.05)	0.49 (±0.20)	0.63 (±0.05)	0	0

BAcc: Balanced Accuracy, MCC: Matthews Correlation Coefficient, AUC: Area Under the ROC Curve

Table S6 Features selected in more than 50% of the shuffle splits of the best models for each subgroup (C, C+M and C+M+BF) when analysing TN tumours.

C	C+M	C+M+BF
Age, SBR_Mitose, Menopause	SBR_Mitose	M_sd, M_contrast, TLG, BF_IDM, BF_correlation, BF_clustershade, Age, SBR_Mitose

BF: Blood Flow, IDM: Inverse Different Moment, M: Metabolism, SBR: Scarff-Bloom-Richardson, TLG: Total Lesion Glycolysis
